# Less-experienced Writers’ Writing Letters: Some Concerns and Recommendations

**DOI:** 10.31662/jmaj.2025-0015

**Published:** 2025-03-14

**Authors:** Shigeki Matsubara

**Affiliations:** 1 Department of Obstetrics and Gynecology, Jichi Medical University, Tochigi, Japan; 2 Department of Obstetrics and Gynecology, Koga Red Cross Hospital, Koga, Japan; 3 Medical Examination Center, Ibaraki Western Medical Center, Chikusei, Japan

**Keywords:** letter, manuscript, paper, skill, training

## Dear Editors,

I commend Dr. Saeki for thoughtfully addressing Letter-writing ^(1)^ and encouraging younger doctors to write papers ^(1), (2)^.

I embrace Letters ^(3), (4)^, similar to Saeki. He repeatedly states: “Letters provide a good platform for ‘less-experienced’ authors.” I am concerned emphasizing “less-experienced” might cause readers’ misunderstanding.

First, readers might consider writing Letters easier than other manuscript types: this is not true. Letters lack “standard” structures like original articles or case reports. I wrote two books on “how to write,” targeting original articles and case reports. I attempted to write “how to write Letters” but could not because no “rules” exist for Letter-writing. Actually, I wrote my first Letter in 2011, which was 24 years after I had written my first original paper in 1987. My personal anecdotes may not apply to everyone; however, I believe “The shorter, the more difficult”.

Second, when Letters addressing a specific article are rejected, “reviving” them is difficult because they are tied to that particular article. This differs from other types of manuscripts, which can often be immediately submitted to another journal. Veterans are accustomed to “rejection”; however, younger authors may become heartbroken when facing “rejection with no next to submit.”

Third, accumulated experience often prompts one to write Letters. I have extensively studied and written about obstetric life-saving surgeries for 30 years. One article described wrong procedures, endangering patients. I could not help but write a disagreement Letter, driven by the necessity born from years of practice. Letters are accessible to all. However, their essence lies in conveying meaningful insights rooted in personal experience, rather than being seen as a part of exercises for honing writing skills.

Content matters for all types of manuscripts, including Letters. Good content benefits readers, irrespective of who, veterans or younger generations, wrote it. My point is: that younger doctors can, and should, write Letters; however, it should be neither because i) Letters may seem easier to write, nor because ii) Letter-writing can be viewed as training for future original articles.

I recommend:

1. Less-experienced writers should focus on writing “Opinion” Letters rather than Agreement/Disagreement Letters ^(3)^ that address a specific article.

2. These pieces should be based on their unique “younger sensitivity.”

Some are talented in writing short pieces, similar to short-story writers. Such a personal gift should be respected. I only suggest for many “ordinary” doctors.

This seasoned doctor eagerly awaits hearing from younger generations, whose voices will enrich academic communication, especially between older and younger generations.

## Article Information

### Conflicts of Interest

None

### Author Contributions

S.M.: Identification of the significance. Manuscript writing.

### Approval by Institutional Review Board (IRB)

Not applicable

### Patient Anonymity

Not applicable.

### Informed Consent

Not applicable.

### Data Availability

Data sharing is not applicable to this article, as no new data were created or analyzed in this study.
